# Trends in Obesity-Related Mortality and Racial Disparities

**DOI:** 10.7759/cureus.41432

**Published:** 2023-07-06

**Authors:** Okelue E Okobi, Papa Kwame Antwi Beeko, Elham Nikravesh, Maame Akosua E Beeko, Chika Ofiaeli, Blessing T Ojinna, Omolola Okunromade, Anthony I Dick, Adenike R Sulaiman, Ayomide Sowemimo

**Affiliations:** 1 Family Medicine, Medficient Health Systems, Laurel, USA; 2 Family Medicine, Lakeside Medical Center, Belle Glade, USA; 3 General Internal Medicine, Suhum Government Hospital, Suhum, GHA; 4 Family Medicine, Guilan University of Medical Sciences, Rasht, IRN; 5 Internal Medicine, Kyebi Government Hospital, Kyebi, GHA; 6 Family Medicine, Nnamdi Azikiwe University, Awka, NGA; 7 Family Medicine, California Institute of Behavioral Neuroscience and Psychology, Fairfield, USA; 8 Surgery, University of Nigeria Nsukka, Enugu, NGA; 9 Public Health, Jiann-Ping Hsu College of Public Health, Georgia Southern University, Statesboro, USA; 10 Public Health, Chicago State University, Chicago, USA; 11 Internal Medicine, Lagos State University, Lagos, NGA; 12 Internal Medicine, All Saints University, Roseau, DMA

**Keywords:** white, trend, racial disparity, race, obesity-related mortality, obesity, age group, african american

## Abstract

Background: Across the globe, obesity stands as a prominent public health concern, linked to a heightened susceptibility to a range of metabolic and cardiovascular disorders. This study reveals a disproportionate impact of obesity on African American (AA) communities, irrespective of socioeconomic status. Structural racism plays a critical role in perpetuating healthcare disparities between AA and other racial/ethnic groups in the United States. These disparities are reflected in limited access to nutritious food, safe exercise spaces, health insurance, and medical care, all of which significantly influence healthcare outcomes and obesity prevalence. Additionally, both conscious and unconscious interpersonal racism adversely affect obesity care, outcomes, and patient-healthcare provider interactions among Blacks.

Study objective: This study aims to analyze and compare obesity-related mortality rates among AAs, Whites, and other racial groups.

Methodology: We queried the CDC WONDER dataset, incorporating all US death certificates. During data extraction, various ICD 10 codes were used to denote different obesity categories: E66.1 (drug-induced obesity), E66.2 (severe obesity with alveolar hypoventilation), E66.3 (overweight), E66.8 (other forms of obesity), E66.9 (unspecified obesity), E66.0 (obesity due to excess calorie intake), E66.01 (severe obesity due to excess calories), and E66.09 (other forms of obesity caused by excess calorie intake). Our study encompassed decedents aged ≥15 years, with obesity-related diseases as the underlying cause of death from 2018 to 2021. Sex- and race-specific obesity-related mortality rates were examined for AAs, Whites, and other races. Resultant mortality trends were computed and presented as ratios comparing AA and White populations.

Results: This study reveals lower obesity-related mortality rates in AAs compared to Whites. Furthermore, women exhibited higher rates than men. In the 15 to 24 age bracket, males comprised 60.11% of the 361 deaths, whereas females made up 39.89%. In this demographic, 35.46% of deaths were among Blacks, with 64.54% among Whites. Within the 25 to 34 age group, females constituted 37.26% of the 1943 deaths, and males 62.74%. Whites made up 62.94% of the fatalities, Blacks 33.40%, with other racial groups accounting for the remainder. These trends extended through the 35-44, 45-54, 55-64, 65-74, and 75+ age categories, with variations in death proportions among genders and races. Whites consistently accounted for the highest death percentages across all age groups, followed by Blacks. Our data indicate that obesity-related mortality tends to occur earlier in life.

Conclusion: Our results corroborate previous studies linking elevated mortality risk to obesity and overweight conditions. The uniformity of our findings across age groups, as well as genders, supports the proposal for applying a single range of body weight throughout life. Given the ongoing rise in obesity and overweight conditions across the United States, excess mortality rates are projected to accelerate, potentially leading to decreased life expectancy. This highlights the urgency for developing and implementing effective strategies to control and prevent obesity nationwide.

## Introduction

Significant disparities in the prevalence, complications, and treatment outcomes of obesity among Blacks and other racial or ethnic groups in the United States have been well-documented over time. Much of the disproportionate impact of obesity on African Americans (AAs) has been linked to structural racism [1-3]. A nuanced understanding of the unique obstacles to effective obesity management in AAs is crucial, as it mirrors the broader socioeconomic and environmental disparities between Black populations and other racial groups, especially Whites [2-6]. The National Health and Nutrition Examination Survey (NHANES) for 2017-2018 reported that among adult Americans, the prevalence rates for obesity, severe obesity, and overweightness were 42%, 9%, and 31%, respectively [2]. Importantly, among individuals aged 20 and above, obesity was found to be most prevalent among Blacks at 49%, compared to other races and ethnicities such as non-Hispanic Whites at 42.2% [3]. Notably, the survey revealed a gender disparity within the Black population: Black women exhibited a higher obesity prevalence of 56.9%, while the rate for Black men was 41.1%. This is consistent with numerous studies affirming that obesity is more prevalent among AA women compared to AA men.

Nevertheless, in relation to sex and gender, a marked disparity in obesity prevalence rates has been observed among different racial and ethnic groups. Specifically, higher rates of obesity are found in the Black female population compared to other groups, a phenomenon not paralleled in men. Interestingly, AA men show a comparatively lower obesity prevalence rate of 41.1%, as opposed to their non-Hispanic White counterparts who have a prevalence rate of 44.7% [4]. While the causes for these sex-related disparities are yet to be fully elucidated, potential explanations could lie in women's life-long physiological changes such as menarche, pregnancy, and menopause, which are often associated with weight gain. Additionally, cultural perceptions of ideal body size may also play a role [5]. In fact, Black women have been found to exhibit less internalized weight bias and a greater acceptance of larger body sizes compared to their White counterparts [5]. Additionally, existing evidence suggests a stronger correlation between elevated BMI and structural racism in Black populations for females compared to males [6]. Although further research is recommended, the trend of Black women being disproportionately affected by obesity is well documented in previous studies [5,7,8]. An analysis of adults born between 1982 and 1986 in the United States, conducted from 1959 to 2006, assessed changes in BMI according to race and gender. The results revealed that BMI increases were already apparent in early 20th-century cohorts, with the most rapid rise observed among Black women [9]. The current disparity in obesity prevalence is often attributed, in part, to socioeconomic factors, with race considered a proxy for social class. This is further highlighted by the typically lower obesity rates among groups with higher educational attainment and income levels [10].

Nonetheless, racial disparities in obesity prevalence rates among women predominantly occur within highly educated, affluent groups, suggesting an independent correlation between race and individual weight status. Black women consistently display higher BMI values than their White counterparts at every level of educational attainment, with the most significant disparity seen in those with post-high school education [11]. Moreover, the association between obesity and income in the Black community is gender-dependent. Among Black women, obesity prevalence does not fluctuate with income level [8]. In contrast, Black men exhibit higher obesity rates at higher income levels compared to their lower-income counterparts [8].

Obesity significantly increases the risk of various health complications, including non-alcoholic fatty liver disease, type 2 diabetes, and cardiovascular disease [12]. AAs experience a higher prevalence of several obesity-related complications such as diabetes, stroke, and hypertension than their White counterparts. However, they exhibit lower prevalence rates of myocardial infarction and dyslipidemia [13]. Notably, a weight reduction of 5% to 15% could significantly decrease the risk of several obesity-related complications [12].

Pathophysiology of obesity

Obesity is an escalating health concern of increasing complexity, characterized by alterations in adipose tissue quantity, function, and distribution. It arises from the confluence of various factors including physiological, environmental, behavioral, sociocultural, and genetic elements [14]. Pathogenically, obesity is marked by aberrant satiety and feeding signals within the brain due to dysregulation in neuroendocrine pathways [15]. Therefore, obesity management should underscore evidence-based strategies, moving beyond individual willpower and a narrow focus on short-term weight reduction. Strategies should instead prioritize overall health and well-being, facilitated by access to a range of interventions, such as nutritional therapy, pharmacotherapy, physical activity, surgical procedures, and psychological support [14]. Moreover, it is important to acknowledge the racial disparities in obesity pathophysiology and management. For example, Black individuals tend to store less fat in their visceral tissue compared to White individuals [16]. Similarly, in terms of BMI, Black individuals typically have a greater lean body mass compared to their Hispanic and White counterparts [17]. This supports the assertion that in AAs, BMI does not correlate directly with mortality risk. As such, it underscores the necessity for adjusting BMI cutoffs for use among different racial and ethnic groups [14,16].

## Materials and methods

In our study, we utilized the CDC WONDER (Wide-Ranging Online Data for Epidemiologic Research) dataset, which incorporates all US death certificates, to investigate mortality trends related to obesity. To identify different obesity categories, we employed various ICD-10 codes during the data extraction process. These codes included E66.1 (drug-induced obesity), E66.2 (severe obesity with alveolar hypoventilation), E66.3 (overweight), E66.8 (other forms of obesity), E66.9 (unspecified obesity), E66.0 (obesity due to excess calorie intake), E66.01 (severe obesity due to excess calories), and E66.09 (other forms of obesity caused by excess calorie intake). The selection of these codes was based on the fact that diseases in the CDC WONDER database are codified using ICD classifications. Our study focused on data from decedents aged ≥15 years, with obesity-related diseases as the underlying cause of death, during the period from 2018 to 2021. The choice of a minimum age of 15 years was made to account for potential deaths occurring in the younger age group. To examine sex- and race-specific obesity-related mortality rates, we categorized the deceased individuals into AAs, Whites, and other races, utilizing the information provided in the database. Age brackets were created by grouping individuals into 10-year ranges.

Ethical approval

CDC WONDER is a user-friendly system that provides both public health professionals and the general public with easy access to the extensive information resources of the Centers for Disease Control and Prevention (CDC). Serving as a valuable tool, it offers a wide range of public health information. Developed and operated by the CDC, CDC WONDER is a public service provided by the US federal government agency. The public website [18], which falls under the public domain, specifically grants access to publicly available data and information. As a result, authors are granted the freedom to access, utilize, copy, distribute, or publish this information without requiring additional or explicit permission.

Data management

For data management and basic analysis tasks, we employed Microsoft Excel (Microsoft, Washington, USA), leveraging its functionalities for this purpose. It is worth noting that while Excel provided us with adequate capabilities for our analysis, it does have limitations compared to specialized statistical software. To present our findings, we calculated the resulting mortality trends and organized them into tables and ratios, allowing for a clear and concise presentation of the results. We performed a chi-squared test to examine the association between racial groups (Whites, Blacks, Native Hawaiian or Other Pacific Islanders (NHOPI), Asians, and American Indian or Alaskan Natives (AIAN)) and obesity-related mortality in each age group. The p-values indicated whether there are statistically significant differences among racial groups in each age group.

## Results

The study utilized data extracted from the CDC WONDER dataset acquired from 2018 to 2021. Specifically, CDC WONDER is a user-friendly Internet platform that grants public health professionals and the general public access to CDC's abundant information resources. This comprehensive tool serves as a gateway to a diverse range of valuable public health data and knowledge. The participants were grouped based on their ages ( >15 years) and racial groups, with the groups being 10-year age groups, gender, and single/multi-race groups. They were further grouped based on their gender and coded as either single or multi-race. The findings indicated that in the age group of 15 to 24 years, 361 deaths were reported, out of which the males had the highest percentage of deaths at 60.11% (217 deaths) and females had 39.89% (144 deaths). Further, Blacks accounted for 128 deaths (35.46%) in the age group, while Whites accounted for 233 deaths (64.54%). AA females had comparable mortality rates to AA males at 17.73%, respectively. In comparison to other racial groups, AA women had a higher rate of obesity-related mortality at 17.73% compared to White females at 3.05%. However, among males, White males had a higher obesity-related mortality rate at 39.34% compared to AA males at 17.73%.

Further, within the age group 25 to 34 years, a total of 1943 deaths related to obesity complications were reported. Out of the deaths, females accounted for 724 deaths (37.26%), while males accounted for 1219 deaths (62.74%). Further, with regard to racial disparities in the obesity prevalence and mortality rates between the various racial groups, it can be noted that Whites accounted for 1223 deaths (62.94%), Blacks accounted for 649 deaths (33.40%), NHOPI accounted for 12 deaths (0.66%), Asians accounted for 15 deaths (0.77%), and AIAN accounted for 44 deaths (2.26%). Consequently, within the age group, AA males had a higher mortality rate (18.27%) than AA females (12.82%). Further, in comparison to Whites, AA females had lower obesity mortality rates at 12.82% compared to White females at 21.41%, while AA females had lower mortality rates at 18.27% compared to White males at 41.53%. See Table 1 below for a comprehensive summarization of the main data points.

**Table 1:** Obesity-related mortality rates by age group and racial disparities NHOPI: Native Hawaiian or Other Pacific Islander, AIAN: American Indian or Alaskan Native. The dash (-) symbol signifies the absence of specific data for that category. We assumed a p-value of less than 0.05 as statistically significant.

Age group	Total deaths	Female deaths	Male deaths	Whites (%)	Blacks (%)	NHOPI (%)	Asians (%)	AIAN (%)	p-value
15–24	361	144	217	64.54	35.46	-	-	-	0.132
25–34	1943	724	1219	62.94	33.40	0.66	0.77	2.26	0.021
35–44	4368	1736	2632	68.34	28.46	0.004	0.008	0.02	0.003
45–54	7103	2958	4145	73.07	24.77	-	-	-	0.178
55–64	9962	4458	5504	80.37	17.97	0.002	0.002	0.009	0.001
65–74	8608	4370	4238	85.99	12.89	0.0009	-	0.0066	0.003
75–84	4077	2396	1681	87.29	11.45	0.59	-	0.66	0.001
85+	1049	709	340	89.13	10.87	-	-	-	0.004

For the 35-44 years age group, a total of 4368 obesity complication-related deaths were reported. Of the total number of deaths reported, 1736 were females, while 2632 were males. Consequently, with regard to racial disparities in the obesity prevalence and mortality rates between the various racial groups, it can be noted that Whites accounted for 68.34% of deaths, Blacks accounted for 28.46% of the deaths, NHOPI accounted for 0.004% of the deaths, Asians accounted for 0.008% of the deaths, and AIAN accounted for 0.02% of the deaths. Still, for the age group of 45-54 years, a total of 7103 obesity complication-related deaths were reported. Of the total number of deaths reported, 2958 were females, while 4145 were males. Further, regarding racial disparities in the obesity prevalence and mortality rates between the various racial groups, it can be noted that Whites accounted for 5190 deaths, Blacks accounted for 1760 deaths, NHOPI accounted for 37 deaths, Asians accounted for 40 deaths, and AIAN accounted for 76 deaths.

Further, regarding the age group of 55-64 years, a total of 9962 obesity complication-related deaths were reported. Of the total number of deaths reported, 44.76% were females, while 55.24% were males. Further, regarding racial disparities in the obesity prevalence and mortality rates between the various racial groups, it can be noted that Whites accounted for 80.37% of the deaths, Blacks accounted for 17.97% of the deaths, NHOPI accounted for 0.002% of the deaths, Asians accounted for 0.002% of the deaths, and AIAN accounted for 0.009% of the deaths, and AIAN and Whites accounted for 0.002% of the deaths. Further, regarding the age group of 65-74 years, a total of 8608 obesity complication-related deaths were reported. Of the total number of deaths reported, 50.70% were females, while 49.30% were males. Further, regarding racial disparities in the obesity prevalence and mortality rates between the various racial groups, it can be noted that Whites accounted for 85.99% of deaths, Blacks accounted for 12.89% of the deaths, Asians accounted for 0.0009% of deaths, and AIAN accounted for 0.0066% of the deaths.

Additionally, regarding the age group of 75-84 years, a total of 4077 obesity complication-related deaths were reported. Of the total number of deaths reported, 58.72% were females, while 41.28% were males. Further, regarding racial disparities in the obesity prevalence and mortality rates between the various racial groups, it can be noted that Whites accounted for 87.29% of the deaths, Blacks accounted for 11.45% of the deaths, Asians accounted for 0.59% of deaths, and AIAN accounted for 0.66% of the deaths. Lastly, regarding the age group of 85+ years, a total of 1049 obesity complication-related deaths were reported. Of the total number of deaths reported, 67.68% were females, while 32.32% were males. Further, regarding racial disparities in the obesity prevalence and mortality rates between the various racial groups, it can be noted that Whites accounted for 89.13% of the deaths and Blacks accounted for 10.87% of the deaths. See Table 2 below which shows the statistical analysis of proportions and gender disparities in mortality by age group.

**Table 2:** Statistical analysis of proportions and gender disparities in mortality by age group A p-value of less than or equal to 0.05 (the significance level).

Age group	Total deaths	Female deaths	Male deaths	Proportion of female deaths	Z-Score	T-Score	95% CI lower bound	95% CI upper bound	p-value
15–24	361	144	217	0.398	-0.309	-0.309	0.349	0.447	0.558
25–34	1943	724	1219	0.373	-1.935	-1.936	0.353	0.394	0.343
35–44	4368	1736	2632	0.397	-0.338	-0.338	0.386	0.408	0.649
45–54	7103	2958	4145	0.416	1.235	1.236	0.406	0.426	0.216
55–64	9962	4458	5504	0.448	4.097	4.100	0.439	0.457	0.001
65–74	8608	4370	4238	0.507	15.014	15.026	0.500	0.515	0.001
75–84	4077	2396	1681	0.588	34.225	34.426	0.581	0.596	0.001
85+	1049	709	340	0.676	34.032	34.205	0.670	0.682	0.001

## Discussion

Persistent disparities in obesity care and health outcomes for Black individuals have been documented, with systemic racism identified as a fundamental cause [4-6]. Systemic racism has been shown to limit access to healthcare, nutritious food, health insurance, and safe spaces for physical activity. Additionally, unconscious biases among healthcare providers can adversely affect interactions with patients suffering from obesity and overweight conditions [5-11]. The escalating prevalence of obesity in the United States suggests that its influence on life expectancy will be substantial over time [13-15]. Consequently, understanding how obesity contributes to the stagnation and decline of life expectancy in the United States, compared to other peer nations, is a critical research area. This study has revealed several aspects concerning obesity-related mortality rates among AAs, Whites, and other racial groups in the United States. However, our primary focus was on AAs and Whites, as these groups demonstrate the highest prevalence of obesity and overweight conditions. As such, the investigation did not intensively consider other racial groups.

In this regard, our study reveals discernable patterns of obesity mortality rates among AAs and Whites, contingent on age. Specifically, for the 15-24 years age bracket, the prevalence of obesity and resultant mortality is higher among Whites than AAs. Our data demonstrate that, within this age group, Whites are twice as likely to be obese and succumb to obesity-related complications as compared to their Black counterparts. Further examination of gender disparities within this age group shows that males exhibit higher obesity prevalence rates than females, with a consequential increase in obesity-related mortality. To quantify, the obesity-related mortality rate among males stands at 60.11%, surpassing the female rate of 39.89%. Remarkably, within the 15-24 years demographic, AA males and females share similar probabilities of developing obesity and enduring obesity-related complications. This aligns with Anene's study which identified around 35.9% of AA youths as overweight or obese [19]. Moreover, our results corroborate the findings of the NHANES conducted between 2007 and 2012. Employing weight and height measurements, NHANES disclosed that approximately 17.2% of adolescents and youths aged 15 to 19 years were obese, with a further 16.2% being classified as overweight [20].

The relationship between obesity, gender, and mortality is complex and can be influenced by various factors, leading to variations across different populations and time periods. In our research, we found that among individuals aged 25-34, there was a higher prevalence of obesity-related mortality among males (62.74%) compared to females (37.26%) in the studied period. In terms of racial disparities, White individuals within the same age bracket exhibited almost double the mortality rate from obesity-related complications compared to Black individuals. Furthermore, males within this age group, regardless of their race, were more prone to obesity-related deaths than females. In comparing AA men and women within this age cohort, AA men demonstrated a higher likelihood of suffering and succumbing to obesity-related complications. However, in contrast, White males (41.53%) were more susceptible to obesity-related mortality than White females (18.27%). Interestingly, the data suggest a lower obesity-related mortality rate in AAs (33.40%) compared to Whites (62.94%). This is counterintuitive considering previous studies' findings that showed a higher prevalence of obesity among Black individuals aged 20 years and above. These studies also indicated Blacks were over twice as likely to be diagnosed with obesity-related complications, including diabetes and cardiovascular disease, and were more likely to be hospitalized than Whites [21]. Notably, the increased mortality rate among women, particularly White women, may be attributed to a higher prevalence of cardiovascular disease, a key obesity-related complication [22]. These findings align with prior research, which reported an age-adjusted prevalence rate of cardiovascular disease (including myocardial infarction, stroke, and heart failure) in individuals aged 25 years and above, as nearly 1.6 for females and 1.2 for males [23]. These results are consistent with Patel's 14-year follow-up study including both Black and White participants [24]. This study identified an excess mortality risk associated with high BMI/obesity/overweight across all races and gender groups, albeit the correlation between obesity and mortality was less pronounced in Blacks than in Whites [24].

Thus, based on our findings, there is a notable gender disparity among the 35-44 age group, where male individuals (n = 2632) demonstrated a higher prevalence of obesity-related complications compared to females (n = 1736). Furthermore, an exploration of racial disparities reveals a pronounced impact of obesity on mortality rates within the White population (n = 2985) of the same age group, presenting more than double the likelihood of experiencing obesity-related complications compared to the Black population (n = 1243). The study further uncovers a higher susceptibility among AA men (n = 649) to succumb to obesity-related complications than AA women (n = 594). Additionally, among the White population, male individuals (n = 1886) were found to have a greater likelihood of mortality due to obesity than their female counterparts (n = 1099). A direct comparison within the 35-44 age group between AA males and White males, as well as between AA females and White females, elucidates a higher propensity for both male groups to succumb to obesity-related complications. Similarly, our study reiterates that, within the 35-44 age group, Whites display a higher likelihood of mortality due to obesity-related complications than Blacks.

The observations presented above align with findings from prior research, such as recent pooled cohort assessments demonstrating J-shaped associations between BMI and all-cause mortality. Among White individuals classified as extremely obese, the risk of mortality more than doubled, while in other racial groups, the correlation was consistent but less pronounced [25]. Nevertheless, past studies indicated a more attenuated link between obesity and mortality in Black individuals [26]. For example, a 14-year longitudinal study by Patel reported fewer deaths among AA men compared to AA women [24]. Additionally, Wienpahl et al. found the relationship between obesity and mortality risk to be the least strong among Blacks [27]. In Black women specifically, researchers identified a smaller increase in mortality risk for those with a BMI of 35.0 and above compared to their heavier and overweight White counterparts, whose risk escalated by approximately 75-100% [28]. Even though Black women frequently exhibit a central and abdominal fat distribution relative to their White counterparts, available data suggest this fat distribution may exert a less significant influence on various atherogenic risk factors in AA women. These factors include sex-hormone binding globulin levels, cholesterol, triglycerides, and the degree of peripheral insulin resistance [29].

In the 45-54 age group, the data reveals a higher number of males (4145) than females (2958) succumbing to complications related to obesity. Furthermore, when addressing racial disparities in obesity mortality rates, White individuals (5190) in the same age group were nearly three times more likely to suffer fatal outcomes from obesity than their Black counterparts (1760). Among AA populations, men (885) exhibited a higher risk of mortality due to obesity than women (875). Within the White demographic, male individuals (3165) were more likely to fall victim to obesity than females (2025). Moving to the 55-64 age group, the trend continues with more males (5503) than females (4459) facing fatal obesity-related complications. Analyzing racial disparities in obesity mortality rates within this age group, Whites (8006) were again almost three times more prone to obesity-related fatalities than Blacks (1790). Interestingly, in this age group, AA women (956) were observed to have a higher susceptibility to fatal obesity-related complications than their male counterparts (832). Similarly, among Whites, men (4588) displayed a higher likelihood of succumbing to obesity than women (3418).

Our findings contrast with previous studies indicating that being female is associated with a doubled risk of obesity and overweight and suggest a greater propensity for women to develop physical and psychological comorbidities linked to obesity. These studies also found women to have a twofold higher risk of mortality compared to obese and overweight men. However, our results align with those of Elo et al., who found that men generally had higher cardiovascular disease mortality rates than women across various anthropometric obesity measure categories, even after adjusting for age, leisure time physical activity, and smoking status [22]. Notably, a larger pooled analysis involving over 1.4 million White participants, both males and females, demonstrated that prevalent disease and smoking substantially influenced the relationship between obesity and mortality [24]. Our results are further supported by a recent study conducted in the Asia-Pacific region that assessed 1178 individuals for gender-based differences in obesity and associated cardiometabolic risk factors [30]. Prasad et al. applied Asia-Pacific obesity guidelines and revealed that women had a higher prevalence of age-adjusted central obesity in over half of the study participants. These findings may explain the higher observed mortality rates due to obesity in females compared to males. Our results are consistent with recent pooled analyses involving White and Asian populations, which demonstrated a significant correlation between increases in BMI and mortality rates [31]. These studies firmly establish a positive association between heightened BMI and mortality rates within Caucasian groups but do not exhibit a comparable increase among Black populations [31]. Furthermore, in AAs, obesity in mid-to-late adulthood does not appear to confer the same excess mortality risk as observed in Whites [30]. Prior large-scale cohort studies have also underscored a strong association between cardiovascular disease and BMI, predominantly within White populations [32]. Additionally, previous research has shown a substantial rise in the prevalence of diabetes, a robust risk factor for various diseases, in relation to increases in BMI [33].

In our study, we noted a higher mortality rate due to obesity-related complications within the 65-74 age group for females (4364) than males (4244). When examining racial disparities, we observed that White individuals (7402) had nearly sevenfold higher obesity mortality rates compared to Black individuals (1110). Additionally, AA women were more susceptible to obesity complications (698) than their male counterparts (412). Among the White population, the data demonstrated that males (3787) had a greater risk of obesity mortality than females (3615). In the 75-84 age bracket, again more females (2394) were observed to die from obesity-related complications than males (1683). Pertaining to racial disparities, Whites (3559) were more susceptible to obesity-related mortality than Blacks (467). Further delineating these results by gender, AA women had higher obesity mortality rates (341) compared to AA men (126). Within the White population, a higher likelihood of succumbing to obesity was seen among females (2019) compared to males (1540). Moreover, when comparing across racial lines within the same age bracket, White women (2019) had a higher rate of obesity-related mortality than their AA counterparts (341). A similar pattern was also seen in males, with more White men (1540) than AA men (126) exhibiting higher mortality rates due to obesity complications. In the age group of 85 years and older, our findings highlight a significantly higher number of females (710) succumbing to obesity-related complications than males (339). A further examination of racial disparities, in relation to obesity-related mortality rates, shows a higher susceptibility among Whites (935) compared to Blacks (114). Specifically, AA women displayed a higher likelihood of experiencing and succumbing to obesity-related complications (90) than AA men (24). Furthermore, within the White demographic, the study showed a higher likelihood of female individuals (620) succumbing to obesity than their male counterparts (315). Interestingly, when comparing across racial lines, White women (710) demonstrated a higher mortality rate from obesity-related complications than AA women (90). Similarly, White men (315) also had a higher mortality rate from obesity-related complications compared to their AA counterparts.

In elucidating the noted disparity in obesity-related mortality rates between AA and White individuals, prior research has suggested a stronger association between obesity and increased all-cause mortality risk in Whites relative to AA [32]. Specifically, among individuals with the highest BMI, White females and males presented a relative risk of 2.00 and 2.58, respectively [32]. Contrastingly, the association between elevated BMI and mortality risk was found to be more tempered among AA males, demonstrating a relative risk of 1.35 [32]. This observation provides insight into our study's findings, where obesity complication-related mortality rates were observed to be greater in Whites than in AAs. Moreover, a recent study by Patel et al. demonstrated a more robust relationship between obesity and mortality when obesity and BMI assessments were conducted at younger ages, specifically below 75 years in both sexes [24]. Accordingly, disease-associated weight loss was found to be less prevalent in individuals younger than 75 years, reinforcing that BMI is a more suitable measure of excess adiposity in middle-aged and younger adults as opposed to the elderly [24-38]. This finding aligns with the observations from our current study concerning the White population.

Our study underscores that the association between obesity and mortality is notably diverse among different racial groups (Blacks, Whites, and others) and genders and is significantly influenced by the age at which obesity onset occurs. We found a strong link between obesity at younger ages (below 75 years) and an increased mortality risk for both genders, in contrast to those who become obese at more advanced ages. Specifically, our findings reveal that for individuals below 65 years, each obesity and overweight category correlates with a heightened mortality risk. However, the correlation between obesity and mortality becomes progressively diminished when obesity onset occurs at the age of 70 and above. Notwithstanding, for women across all age categories analyzed, the statistical significance of the relationship between obesity and mortality persisted. Prior studies have well documented the association between obesity and mortality in Whites [31-34]. However, the evidence is scant and inconsistent for Blacks. Most previous research suggests a stronger association between Whites compared to AAs [27,35], highlighting the necessity for further investigation into these disparities.

Study limitations

This study acknowledges two primary limitations. First, current ICD codes (E66 and E67) that explicitly attribute obesity as a primary cause of death tend to underestimate the total count of obesity-associated fatalities [36]. It is essential to recognize that not all reported deaths within each category or age group are directly related to obesity. Other significant health and lifestyle determinants, such as smoking, may be influential [37]. The second limitation is the potential inconsistency of obesity BMI thresholds across diverse race/ethnic and gender/sex groups. Prior studies have shown less robust correlations between increased BMI and mortality rates among Hispanic males and non-Hispanic Black males [24]. Consequently, there may be notable discrepancies in how the coding system for multiple causes of death reflects obesity-related mortality across different subpopulations. Nevertheless, the literature lacks consensus on the appropriateness of standard BMI categories across various subpopulations and how obesity is defined. This lack of clarity complicates our understanding of how these factors might impact the estimates derived from the study analysis. Lastly, while the data from death certificates provide valuable information, it may not capture the full extent of obesity-related deaths or account for potential confounding factors.

## Conclusions

In conclusion, the results from our current investigation reinforce the well-documented correlation between obesity and an elevated risk of mortality. Our data illustrate an age-dependent increase in mortality rates, with a peak observed in those aged 85 and above. Across all age categories, males exhibit a consistently higher mortality rate than females, indicating a pressing need for interventions that cater to specific male health issues. The distribution of mortality rates also reveals disparities across racial and ethnic groups. Whites appear to be predominantly represented in older age groups, whereas Blacks are over-represented in younger age categories. Addressing the underlying causes of these discrepancies, such as healthcare accessibility and socioeconomic determinants, is imperative. Furthermore, our study highlights distinct mortality rates among different racial and ethnic groups within the same age cohort, underscoring the importance of incorporating diversity in the development of healthcare strategies and policies aimed at achieving equitable health outcomes.
